# Supplementary figures and images for: Association between triglyceride-glucose derived indices with cardiometabolic multimorbidity: Findings from the Atherosclerosis Risk in Communities study
Source: PLoS One. 2025 Dec 30;20(12):e0339646. doi: 10.1371/journal.pone.0339646 (PMC12752969; doi:10.1371/journal.pone.0339646)

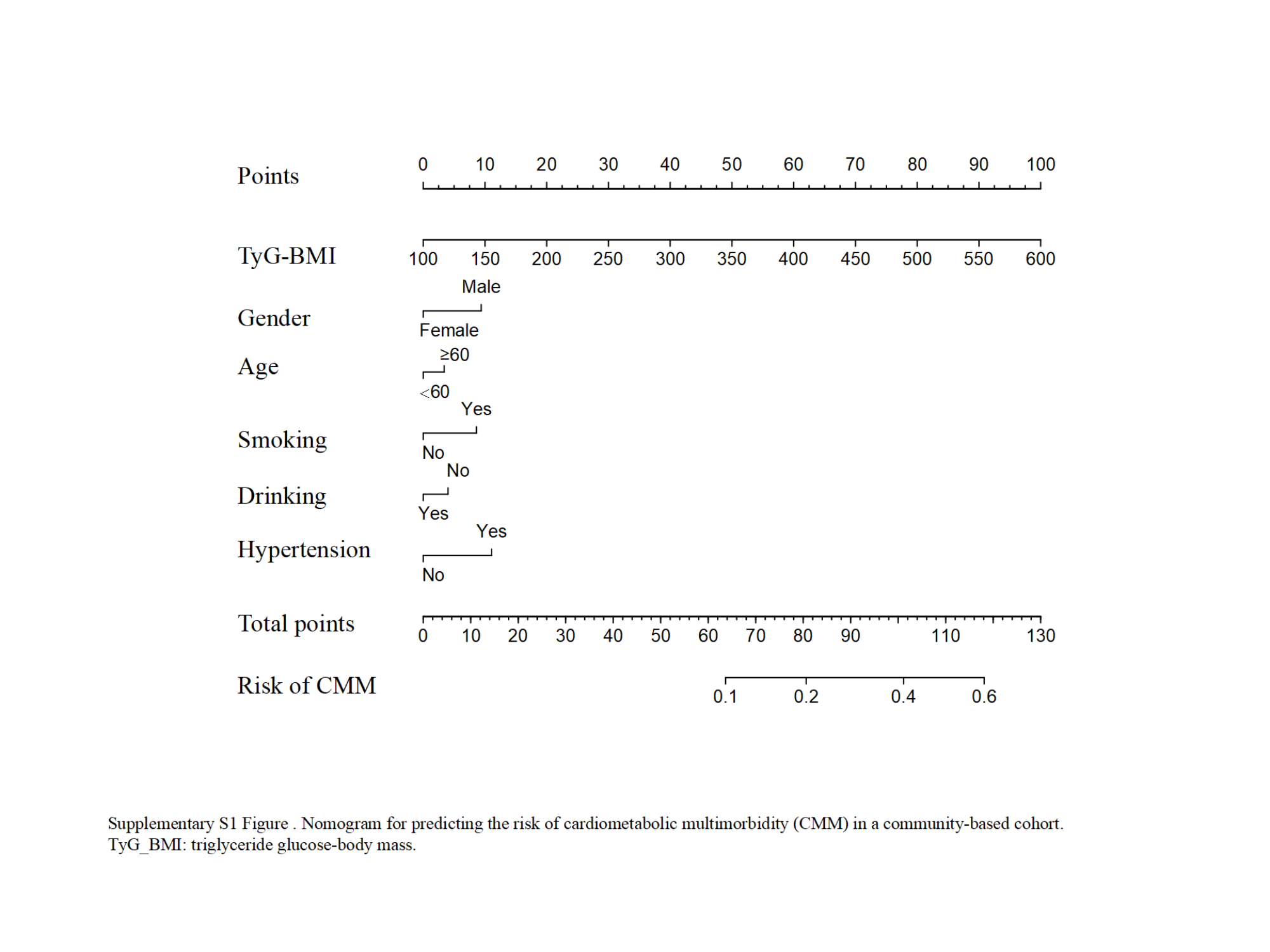

Supplement: S1 Fig — (TIFF) [file pone.0339646.s001.tiff]
