# Supplementary material for: Association between triglyceride-glucose derived indices with cardiometabolic multimorbidity: Findings from the Atherosclerosis Risk in Communities study
Source: PLoS One. 2025 Dec 30;20(12):e0339646. doi: 10.1371/journal.pone.0339646 (PMC12752969; doi:10.1371/journal.pone.0339646)
Supplement: Attachment — Submitted filename: manuscript (2).docx [file pone.0339646.s002.docx]

**Association between triglyceride-glucose derived indices with cardiometabolicmultimorbidity: Findings from the Atherosclerosis Risk in Communities study**

**Short title: TyG-derived indices and cardiometabolic multimorbidity in the ARIC study**

Yi Liu, RN, BSc^1^; Dongze Li, MD, PhD^1^; Jing Yu, MBBS^1^; Yongli Gao, RN, BS^1^; Wei Zhang, RN, BS^1^; Menglin Tang, RN, BS^2^

^1^Department of Emergency Medicine, West China Hospital, Sichuan University/West China School of Nursing, Sichuan University, 37 Guoxue Lane, Chengdu, 610041, Sichuan, China.

^2^Department of Cardiac Surgery, West China Hospital, Sichuan University/West China School of Nursing, Sichuan University, 37 Guoxue Lane, Chengdu, 610041, Sichuan, China.

**Corresponding author**

Prof. Menglin Tang, BS

Department of Cardiac Surgery, West China Hospital, Sichuan University, 37 Guoxue Road, Chengdu 610041, Sichuan, China.

Tel: +86-28-85422461, Fax: +86-28-85422288

E-mail: menglin_tang@163.com

**Abstract**

**Background:** Cardiometabolic multimorbidity (CMM), characterized by the co-occurrence of diabetes mellitus (DM), stroke, and coronary heart disease (CHD), poses a significant global health burden owing to its association with elevated mortality, reduced functional capacity, and increased healthcare costs. Despite its clinical importance, the role of insulin resistance (IR) and its surrogates, particularly triglyceride-glucose (TyG) indices combined with anthropometric measures, in predicting CMM remains underexplored.

**Methods:** To quantify the association between TyG-derived indices and incident CMM, we conducted a multivariate logistic regression analysis within the Atherosclerosis Risk in Communities (ARIC) cohort, deriving adjusted odds ratios (ORs) and 95% confidence intervals (CIs). Nonlinear associations were investigated using restricted cubic spline modeling, whereas diagnostic accuracy was evaluated using area under the curve (AUC) calculations from receiver operating characteristic (ROC) analyses.

**Results:** Nonlinear relationships were observed between TyG-body mass index (TyG-BMI) and TyG-WHtR, whereas TyG and TyG-waist circumference exhibited linear trends. TyG-BMI demonstrated the strongest association with CMM risk, showing a 1.61-fold increase per standard deviation (adjusted OR: 1.61; 95% CI: 1.48–1.73) and a 5.67-fold higher risk in the highest versus the lowest quartile. Predictive performance analysis revealed that TyG-BMI was the most discriminative marker (AUC: 0.684; 95% CI: 0.664–0.705).

**Conclusions:** TyG-BMI emerged as a robust predictor of CMM, highlighting the synergistic effects of IR and adiposity. The nonlinear risk escalation suggests threshold-dependent mechanisms, emphasizing its utility in early risk stratification.

**1 Introduction**

Cardiometabolic multimorbidity (CMM) is the co-occurrence of two or more cardiometabolic diseases [1], including diabetes mellitus (DM), stroke, and coronary heart disease (CHD). It is widely reported to be associated with exponentially increased mortality, diminished functional capacity, and elevated healthcare costs [1–4]. Driven by extended life expectancies, the increasing prevalence of CMM poses substantial challenges to individuals and healthcare systems [5]. However, previous research has predominantly focused on individual cardiometabolic conditions, with limited exploration of complex multimorbidity patterns [6–8], leaving the risk factors and underlying mechanisms of CMM poorly characterized.

Insulin resistance (IR), a physiological state marked by reduced responsiveness to insulin, plays a pivotal role in the initiation, progression, and pathogenesis of cardiometabolic diseases [9–11]. Despite their clinical relevance, the complexity and cost of direct IR measurements hinder their applicability in large-scale clinical and epidemiological studies [12]. Emerging evidence suggests that triglyceride-glucose (TyG)-related indices may serve as practical surrogate markers of IR in routine clinical practice [13–15]. These indices have been associated with mortality, cardiovascular events (e.g., myocardial infarction and stroke), and metabolic disorders across diverse populations [16–18]. However, conclusive evidence regarding their comparative utility in predicting the incidence of CMM remains scarce.

To address these gaps, we integrated TyG with anthropometric measures (body weight, waist circumference, and height) to construct four clinically accessible IR surrogates: TyG, TyG-body mass index (TyG-BMI), TyG-waist circumference (TyG-WC), and TyG-waist-to-height ratio (TyG-WHtR). Our objective was to investigate their association with incident CMM, identify the optimal predictive biomarker for this complex multimorbidity phenotype, and offer actionable strategies for early CMM risk stratification.

**2 Materials and Methods**

**2.1 Study design and population**

The Atherosclerosis Risk in Communities (ARIC) study [19] is a longitudinal cohort investigation designed to evaluate the risk determinants of atherosclerotic progression and associated cardiovascular pathologies. Between 1987 and 1989, the research protocol enrolled 15,792 participants aged 45–65 years, recruited from four distinct U.S. communities: northwestern suburban Minneapolis (Minnesota), Washington County (Maryland), Forsyth County (North Carolina), and Jackson (Mississippi). Comprehensive baseline evaluations (designated visit 1) included a systematic collection of sociodemographic characteristics, medical histories, and clinical parameters. Subsequent follow-up evaluations were conducted at four intervals: visits 2 (1990–1992), 3 (1993–1995), 4 (1996–1998), and 5 (2011–2013). This study received ethical approval from the institutional review boards of all participating centers, and written informed consent was obtained from each participant prior to their participation. The present analysis was obtained from West China Hospital of Sichuan University, and the de-identified dataset was accessed in February 2024.

This analytical cohort utilized visit 1 as the baseline reference, incorporating all 15,792 initially enrolled subjects. Individuals with pre-existing CHD, stroke, or DM (n = 2,799) as well as those with incomplete covariate data (n = 543) were excluded. Thus, the final analytical sample consisted of 12,450 participants.

**2.2 Ascertainment of CMM**

The primary outcome was the incidence of CMM, defined as the concurrent diagnosis of two or more chronic conditions, including DM, stroke, and CHD. The identification of CHD and stroke events followed a standardized protocol that involved annual telephone interviews with participants or proxies, structured study visits, a systematic review of cardiovascular disease discharge records from local hospitals, and mortality certificate analyses. Diabetes was ascertained based on three complementary criteria: (1) self-reported physician diagnosis, (2) documented use of glucose-lowering medications, and (3) laboratory confirmation of glycated hemoglobin (HbA1c) levels ≥6.5% measured during at least two separate clinical encounters.

**2.3 Assessment of triglyceride-glucose-related obesity indices**

Blood samples were collected after an 8-hour fasting period and analyzed in centralized clinical chemistry and lipid laboratories. Anthropometric measurements, including body weight, height, and waist circumference, were obtained during physical examinations conducted at mobile examination centers. Participants were stratified into quartiles (Q1–Q4) based on the TyG, TyG-waist, TyG-WHtR, and TyG-BMI indices. Q1 served as the reference group for comparative analyses.

TyG, TyG-BMI, TyG-waist, and TyG-WHtR were calculated according to the following formulas: (1) TyG = ln [triglycerides (mg/dl) × glucose (mg/dl)/2]; (2) TyG-BMI = TyG × body mass (kg)/height^2^ (m^2^); (3) TyG-WHtR = TyG × waist circumference/height; (4) TyG-waist = TyG × waist circumference.

**2.4 Covariates assessment**

Baseline characteristics, including demographic variables, clinical parameters, and comorbidities, were systematically documented. Smoking status was defined as never smoked or smoked. Lipid profiles were quantified using validated methodologies. Low-density lipoprotein cholesterol (LDL-C) concentrations were calculated using the Friedewald equation, and high-density lipoprotein cholesterol (HDL-C) measurements were obtained using enzymatic colorimetric techniques following established protocols. Hypertensive status was operationally defined by meeting any of three criteria: (1) systolic blood pressure ≥140 mmHg on standardized measurement, (2) diastolic blood pressure ≥90 mmHg, or (3) self-reported antihypertensive medication use within the preceding 14-day period.

**2.5 Statistical analysis**

Continuous variables were presented as either the median (interquartile range) for non-normally distributed data or as mean ± standard deviation for parametric distributions, with between-group comparisons conducted using Mann–Whitney U test, Kruskal–Wallis H test, or analysis of variance as appropriate. Categorical variables were expressed as frequencies (percentages) and analyzed using the chi-square test.

Multivariate logistic regression analyses were performed to evaluate the associations between cognitive performance metrics and incident atrial fibrillation, with the results reported as odds ratios (ORs) and corresponding 95% confidence intervals (CIs). The regression models were sequentially adjusted for potential confounders across three hierarchical levels: demographic characteristics (sex, ethnicity, and age), lifestyle factors (drinking and smoking status), combined clinical biomarkers (total cholesterol, HDL, LDL, and triglycerides), and history of hypertension. Parallel linear regression modeling was implemented to quantify the relationships between triglyceride-glucose-derived indices and CMM.

Statistical significance was established at a two-tailed p-value of 0.05. Statistical computations were performed using SPSS Statistics (version 26.0; IBM Corporation, Armonk, NY, USA) and R statistical software 4.4.2 (R Foundation for Statistical Computing, Vienna, Austria).

**3 Results**

**3.1 Baseline characteristics**

The study comprised 12,450 participants with a mean age of 53.96 ± 5.73 years. Demographic characteristics revealed 5,531 males (44.4%), 9,611 white individuals (77.2%), and a mean BMI of 27.25 ±5.13 kg/m². Hypertension history was documented in 3,168 participants (25.4%).

The study was stratified into two cohorts based on CMM development status. Comparative analyses between the groups demonstrated significant disparities in baseline parameters, including age, sex distribution, BMI, ethnicity, smoking status, drinking status, history of hypertension, and lipid profiles (triglycerides, total cholesterol, HDL-C, and LDL-C) as detailed in Table 1.

**Table 1. Characteristics of adults by cardiometabolic multimorbidity.**

| **Characteristic** | **Non-CMM** | **CMM** | **p-value** | q-value^1^ |
| --- | --- | --- | --- | --- |
|  | (n = 11,889) | (n = 561) |  |  |
| African Americans, n (%) | 2,670 (22%) | 169 (30%) | <0.001 | <0.001 |
| Male sex, n (%) | 5,222 (44%) | 309 (55%) | <0.001 | <0.001 |
| Age, years | 54 (49, 59) | 55 (50, 60) | <0.001 | <0.001 |
| Body mass index, kg/m^2^ | 26.4 (23.6, 29.7) | 28.8 (26.1, 32.3) | <0.001 | <0.001 |
| Drinking, n (%) | 7,048 (59%) | 301 (54%) | 0.008 | 0.009 |
| Smoking, n (%) | 6,897 (58%) | 378 (67%) | <0.001 | <0.001 |
| Hypertension, n (%) | 2,931 (25%) | 237 (42%) | <0.001 | <0.001 |
| Blood glucose, mmol/l | 5.44 (5.11, 5.77) | 5.88 (5.50, 6.27) | <0.001 | <0.001 |
| Triglycerides, mg/dl | 1.19 (0.86, 1.66) | 1.46 (1.06, 2.05) | <0.001 | <0.001 |
| Total cholesterol, mg/dl | 5.46 (4.81, 6.15) | 5.66 (5.04, 6.34) | <0.001 | <0.001 |
| HDL, mg/dl | 1.29 (1.05, 1.62) | 1.10 (0.92, 1.37) | <0.001 | <0.001 |
| LDL, mg/dl | 3.47 (2.86, 4.15) | 3.75 (3.18, 4.36) | <0.001 | <0.001 |
| TyG | 8.55 (8.21, 8.91) | 8.84 (8.49, 9.20) | <0.001 | <0.001 |
| TyG_BMI | 226 (199, 261) | 258 (231, 290) | <0.001 | <0.001 |
| TyG_waist | 824 (744, 909) | 853 (766, 931) | <0.001 | <0.001 |
| TyG_WhtR | 4.84 (4.41, 5.37) | 5.02 (4.57, 5.55) | <0.001 | <0.001 |
| Death, n (%) | 5,062 (43%) | 305 (54%) | <0.001 | <0.001 |

^1^False discovery rate correction for multiple testing

CMM, cardiometabolic multimorbidity; HDL, High-Density Lipoprotein; LDL, Low-Density Lipoprotein; TyG, triglyceride glucose; TyG_BMI, triglyceride glucose-body mass index;TyG_waist, triglyceride glucose-waist circumference; TyG_WHtR, triglyceride glucose-waist to height ratio.

**3.2 TyG-derived indices and CMM**

Multivariate-adjusted logistic regression models were used to investigate longitudinal associations between the four TyG-derived indices and incident CMM risk. As shown in Table 2, a per standard increase in the TyG index, TyG-BMI, TyG-waist circumference, and TyG-WHtR corresponded to adjusted ORs of 1.51 (95% CI: 1.38–1.65), 1.61 (95% CI: 1.48–1.73), 1.15 (95% CI: 1.06–1.25), and 1.16 (95% CI: 1.07–1.26) (Model 3, p < 0.01).

**Table 2. Associations between the triglyceride glucose index and triglyceride glucose-related obesity indices and the cardiometabolic multimorbidity risks.**

| **Exposure** | **Model 1** | **p-value** | **Model 2** | **p-value** | **Model 3** | **p-value** |
| --- | --- | --- | --- | --- | --- | --- |
| **TyG** |  |  |  |  |  |  |
|  | **1.73 (1.59, 1.88)** | <0.001 | **1.69 (1.55, 1.84)** | <0.001 | **1.51 (1.38, 1.65)** | <0.001 |
| Q1 | 1 |  | 1 |  | 1 |  |
| Q2 | **1.47 (1.09, 1.99)** | 0.0118 | **1.40 (1.03, 1.89)** | 0.0299 | **1.40 (1.03, 1.90)** | 0.0305 |
| Q3 | **2.85 (2.17, 3.75)** | <0.001 | **2.61 (1.98, 3.44)** | <0.001 | **2.63 (2.00, 3.48)** | <0.001 |
| Q4 | **4.21 (3.20, 5.53)** | <0.001 | **3.60 (2.73, 4.76)** | <0.001 | **3.66 (2.75, 4.88)** | <0.001 |
|  |  | <0.001 |  | <0.001 |  | <0.001 |
| **TyG_BMI** |  |  |  |  |  |  |
|  | **1.65 (1.53, 1.77)** | <0.001 | **1.69 (1.57, 1.81)** | <0.001 | **1.61 (1.48, 1.73)** | <0.001 |
| Q1 | 1 |  | 1 |  | 1 |  |
| Q2 | **2.14 (1.47, 3.13)** | <0.001 | **1.92 (1.31, 2.81)** | 0.0008 | **1.91 (1.30, 2.79)** | 0.0009 |
| Q3 | **4.93 (3.49, 6.96)** | <0.001 | **4.20 (2.96, 5.94)** | <0.001 | **4.12 (2.91, 5.84)** | <0.001 |
| Q4 | **7.16 (5.10, 10.04)** | <0.001 | **6.01 (4.27, 8.47)** | <0.001 | **5.67 (4.02, 8.01)** | <0.001 |
|  |  | <0.001 |  | 0.001 |  | 0.002 |
| **TyG_waist** |  |  |  |  |  |  |
|  | **1.20 (1.10, 1.30)** | <0.001 | **1.17 (1.08, 1.27)** | <0.001 | **1.15 (1.06, 1.25)** | <0.001 |
| Q1 | 1 |  | 1 |  | 1 |  |
| Q2 | 1.24 (0.96, 1.61) | 0.1015 | 1.20 (0.92, 1.56) | 0.1753 | 1.18 (0.91, 1.54) | 0.2147 |
| Q3 | **1.39 (1.08, 1.80)** | 0.0114 | **1.31 (1.02, 1.70)** | 0.0379 | **1.29 (1.00, 1.68)** | 0.0497 |
| Q4 | **1.76 (1.38, 2.25)** | <0.001 | **1.61 (1.26, 2.06)** | 0.0002 | **1.59 (1.24, 2.04)** | 0.0003 |
|  |  | <0.001 |  | <0.001 |  | <0.001 |
| **TyG_WhtR** |  |  |  |  |  |  |
|  | **1.19 (1.10, 1.29)** | <0.001 | **1.18 (1.09, 1.28)** | <0.001 | **1.16 (1.07, 1.26)** | <0.001 |
| Q1 | 1 |  | 1 |  | 1 |  |
| Q2 | **1.32 (1.02, 1.71)** | 0.0382 | 1.26 (0.97, 1.64) | 0.0807 | 1.25 (0.96, 1.63) | 0.0959 |
| Q3 | **1.46 (1.13, 1.89)** | 0.0035 | **1.38 (1.07, 1.79)** | 0.0137 | **1.37 (1.06, 1.77)** | 0.0177 |
| Q4 | **1.80 (1.41, 2.31)** | <0.001 | **1.66 (1.29, 2.13)** | <0.001 | **1.64 (1.28, 2.11)** | <0.001 |
|  |  | <0.001 |  | <0.001 |  | <0.001 |

Model 1: Unadjusted model.

Model 2: Adjusted for age, sex, ethnicity, and history of hypertension.

Model 3: Model 2 further adjusted for smoking, drinking, triglycerides, low-density lipoprotein, and high-density lipoprotein.

TyG, triglyceride glucose; TyG_BMI, triglyceride glucose-body mass index; TyG_waist, triglyceride glucose-waist circumference; TyG_WHtR, triglyceride glucose-waist to height ration

### 3.3 Nonlinear associations of TyG-related indices with CMM

Restricted cubic spline regression analyses were conducted to flexibly model and visualize the dose–response relationships between TyG-derived indices and the risk of new-onset CMM risk. TyG and TyG-waist displayed linear relationships with CMM (P_nonlinear = 0.531; P_nonlinear = 0.258). Conversely, TyG-BMI and TyG-WHtR displayed nonlinear relationships with CMM risk (P_nonlinear < 0.001, Figure 2).

### 3.4 Predictive performance of the TyG-related indices for CMM

Figure 3 shows the comparative predictive performances of the four TyG-derived metabolic indices in CMM risk stratification. TyG-BMI demonstrated the highest discriminative capacity, achieving an area under the curve (AUC) of 0.684 (95% CI: 0.664–0.705). This was followed by the TyG index, which yielded an AUC of 0.656 (95% CI: 0.633–0.678).

**4 Discussion**

This cross-sectional investigation elucidated the association between TyG-derived indices (TyG, TyG-BMI, TyG-WHtR, and TyG-waist) and CMM in American adults. Our analyses revealed that incident CMM has a linear relationship with TyG levels and waist circumference, contrasting with the nonlinear positive associations observed for TyG-BMI and TyG-waist. To ensure robust conclusions, three hierarchical regression models were constructed by sequentially adjusting for demographic covariates (age, sex, and ethnicity), clinical parameters (history of hypertension, triglyceride, low-density lipoprotein, and high-density lipoprotein levels), and behavioral variables (smoking and alcohol consumption). The TyG-BMI index demonstrated superior predictive accuracy for incident CMM compared with other indices, with each unit increase corresponding to a 1.61-fold elevated risk (adjusted OR: 1.61; 95% CI: 1.48–1.73). Notably, participants in the highest TyG-BMI quartile exhibited a 5.67-fold increase in CMM risk compared with those in the lowest quartile (Q4 vs. Q1).

CMM is a prevalent pattern of multimorbidity associated with substantially reduced life expectancy [1]. However, risk factors for CMM remain unclear. The TyG index [8,14,16], a validated surrogate marker of IR, has emerged as an independent predictor of cardiovascular outcomes. Data from the Prospective Urban Rural Epidemiology cohort established the prognostic value of TyG for cardiovascular mortality, major adverse cardiovascular events (myocardial infarction/stroke), and type 2 diabetes incidence [7]. Parallel findings from a large Korean cohort reinforce the predictive capacity of TyG for CVD risk stratification [14]. However, the association between TyG and CMM remains unclear.

Anthropometric indices (BMI, waist circumference [WC], and WHtR) serve as established proxies for obesity-mediated metabolic dysregulation [20–22]. Compelling evidence links obesity severity with graded increases in cardiometabolic risk; overweight individuals demonstrate doubled risk compared to their normal-weight counterparts, escalating to ten-fold elevations in severe obesity [23]. Recent investigations [24–26] consistently demonstrate enhanced CVD risk prediction when TyG is combined with obesity indices, findings further supported by a Chinese cohort study [27]. The conclusions drawn from these studies are generally consistent with our findings regarding the predictive power of TyG-derived indices for CMM, in which TyG-BMI plays a key role in predicting CMM risk.

Mechanistically, adipose tissue inflammation and mitochondrial dysfunction in obesity-induced IR may explain this association. Obese individuals with IR exhibit upregulated proinflammatory gene expression, diminished mitochondrial oxidative capacity, reduced AMP kinase activity, and elevated oxidative stress [28,29]. Notably, metabolically unhealthy normal-weight individuals paradoxically demonstrate higher cardiometabolic risks than their metabolically healthy obese counterparts, underscoring the complex interplay between adiposity distribution and metabolic homeostasis [30]. Our findings support the use of TyG combined with obesity indices as an integrated biomarker for CMM, although mechanistic studies are needed to clarify the underlying pathways.

The limitations of this study warrant further investigation. First, residual confounding factors persisted despite comprehensive adjustments for covariates. Second, the reliance on self-reported physician diagnoses for CMM classification introduces a potential bias in classification. Third, the U.S. community-based cohort limits generalizability to other ethnicities/nationalities. Future multinational cohort studies employing standardized diagnostic criteria are needed to validate these findings.

**5 Conclusion**

This study established TyG-BMI as a robust predictor of CMM development, with nonlinear risk escalation patterns suggesting threshold effects. The integrative nature of TyG, combined with obesity indices, captures synergistic metabolic dysregulation pathways, potentially providing targeted preventive strategies. Subsequent research should focus on elucidating the molecular interplay between adipose dysfunction, IR, and multimorbidity.

**Acknowledgments**

The authors appreciate the patients’ participation and contribution to this research in the ARIC study.

**Author Contributions**

Conceptualization: Dongze Li

Data curation: Yongli Gao

Formal analysis: Yi Liu

Methodology: Yi Liu

Project administration: Wei Zhang

Software: Yi Liu

Supervision: Jing Yu

Visualization: Yi Liu

Writing - original draft: Yi Liu

Writing - review & editing: Menglin Tang

**Competing interests**

The authors declare no competing interests.

**Ethics approval and consent to participate**

Not applicable. This study utilized data from publicly available databases, which do not contain any identifiable information about individual persons.

**References**

1.	Emerging Risk Factors Collaboration, Di Angelantonio E, Kaptoge S, Wormser D, Willeit P, Butterworth AS, et al. Association of Cardiometabolic Multimorbidity With Mortality. JAMA. 2015;314: 52–60. doi:10.1001/jama.2015.7008

2.	McKee M, Dunnell K, Anderson M, Brayne C, Charlesworth A, Johnston-Webber C, et al. The changing health needs of the UK population. Lancet. 2021;397: 1979–1991. doi:10.1016/S0140-6736(21)00229-4

3.	Pietzner M, Stewart ID, Raffler J, Khaw K-T, Michelotti GA, Kastenmüller G, et al. Plasma metabolites to profile pathways in noncommunicable disease multimorbidity. Nat Med. 2021;27: 471–479. doi:10.1038/s41591-021-01266-0

4.	Jiao YY, Wang LS, Jiang HR, Jia XF, Wang ZH, Wang HJ, et al. [Epidemiological characteristics and trends of cardiometabolic risk factors in residents aged 18-64 years in 15 provinces of China]. Zhonghua Liu Xing Bing Xue Za Zhi. 2022;43: 1254–1261. doi:10.3760/cma.j.cn112338-20220228-00155

5.	Barnett K, Mercer SW, Norbury M, Watt G, Wyke S, Guthrie B. Epidemiology of multimorbidity and implications for health care, research, and medical education: a cross-sectional study. Lancet. 2012;380: 37–43. doi:10.1016/S0140-6736(12)60240-2

6.	Zhang H, Tu Z, Liu S, Wang J, Shi J, Li X, et al. Association of different insulin resistance surrogates with all-cause and cardiovascular mortality among the population with cardiometabolic multimorbidity. Cardiovasc Diabetol. 2025;24: 33. doi:10.1186/s12933-025-02576-0

7.	Rafiee H, Mohammadifard N, Nouri F, Alavi Tabatabaei G, Najafian J, Sadeghi M, et al. Association of triglyceride glucose index with cardiovascular events: insights from the Isfahan Cohort Study (ICS). Eur J Med Res. 2024;29: 135. doi:10.1186/s40001-024-01728-4

8.	Liang S, Wang C, Zhang J, Liu Z, Bai Y, Chen Z, et al. Triglyceride-glucose index and coronary artery disease: a systematic review and meta-analysis of risk, severity, and prognosis. Cardiovasc Diabetol. 2023;22: 170. doi:10.1186/s12933-023-01906-4

9.	Ascaso JF, Pardo S, Real JT, Lorente RI, Priego A, Carmena R. Diagnosing insulin resistance by simple quantitative methods in subjects with normal glucose metabolism. Diabetes Care. 2003;26: 3320–3325. doi:10.2337/diacare.26.12.3320

10.	Teymoori F, Jahromi MK, Ahmadirad H, Daftari G, Mokhtari E, Farhadnejad H, et al. The association of dietary and lifestyle indices for insulin resistance with the risk of cardiometabolic diseases among Iranian adults. Sci Rep. 2023;13: 6224. doi:10.1038/s41598-023-33505-4

11.	Ascaso JF, Pardo S, Real JT, Lorente RI, Priego A, Carmena R. Diagnosing insulin resistance by simple quantitative methods in subjects with normal glucose metabolism. Diabetes Care. 2003;26: 3320–3325. doi:10.2337/diacare.26.12.3320

12.	Guerrero-Romero F, Simental-Mendía LE, González-Ortiz M, Martínez-Abundis E, Ramos-Zavala MG, Hernández-González SO, et al. The product of triglycerides and glucose, a simple measure of insulin sensitivity. Comparison with the euglycemic-hyperinsulinemic clamp. J Clin Endocrinol Metab. 2010;95: 3347–3351. doi:10.1210/jc.2010-0288

13.	Shi S, Song Y, Liu Z, He J, Zheng Z, Song C, et al. The association of the triglyceride-glucose index with the risk of atrial fibrillation: Analysis of the UK Biobank. Nutr Metab Cardiovasc Dis. 2024; 103826. doi:10.1016/j.numecd.2024.103826

14.	Moon JH, Kim Y, Oh TJ, Moon JH, Kwak SH, Park KS, et al. Triglyceride-Glucose Index Predicts Future Atherosclerotic Cardiovascular Diseases: A 16-Year Follow-up in a Prospective, Community-Dwelling Cohort Study. Endocrinol Metab (Seoul). 2023;38: 406–417. doi:10.3803/EnM.2023.1703

15.	Zhao R, Ji X, Wang F. Association of triglyceride glucose-related obesity indices with sarcopenia among U.S. adults: a cross-sectional study from the National Health and Nutrition Examination Survey. Sci Rep. 2025;15: 574. doi:10.1038/s41598-024-84278-3

16.	Liu X, Abudukeremu A, Jiang Y, Cao Z, Wu M, Ma J, et al. U-shaped association between the triglyceride-glucose index and atrial fibrillation incidence in a general population without known cardiovascular disease. Cardiovasc Diabetol. 2023;22: 118. doi:10.1186/s12933-023-01777-9

17.	Huang H, Xiong Y, Zhou J, Tang Y, Chen F, Li G, et al. The predictive value of estimated glucose disposal rate and its association with myocardial infarction, heart failure, atrial fibrillation and ischemic stroke. Diabetes Obes Metab. 2025. doi:10.1111/dom.16132

18.	Dai M, Li D, Yu J, Liu Y, Zhang Q, Zhang W, et al. Association between the triglyceride-glucose index and silent myocardial infarction in the general population. Sci Rep. 2025;15: 8571. doi:10.1038/s41598-025-92779-y

19.	Wright JD, Folsom AR, Coresh J, Sharrett AR, Couper D, Wagenknecht LE, et al. The ARIC (Atherosclerosis Risk In Communities) Study: JACC Focus Seminar 3/8. J Am Coll Cardiol. 2021;77: 2939–2959. doi:10.1016/j.jacc.2021.04.035

20.	Mokdad AH, Ford ES, Bowman BA, Dietz WH, Vinicor F, Bales VS, et al. Prevalence of obesity, diabetes, and obesity-related health risk factors, 2001. JAMA. 2003;289: 76–79. doi:10.1001/jama.289.1.76

21.	Aung K, Lorenzo C, Hinojosa MA, Haffner SM. Risk of Developing Diabetes and Cardiovascular Disease in Metabolically Unhealthy Normal-Weight and Metabolically Healthy Obese Individuals. J Clin Endocrinol Metab. 2014;99: 462–468. doi:10.1210/jc.2013-2832

22.	Xia M-F, Chen Y, Lin H-D, Ma H, Li X-M, Aleteng Q, et al. A indicator of visceral adipose dysfunction to evaluate metabolic health in adult Chinese. Sci Rep. 2016;6: 38214. doi:10.1038/srep38214

23.	Kivimäki M, Kuosma E, Ferrie JE, Luukkonen R, Nyberg ST, Alfredsson L, et al. Overweight, obesity, and risk of cardiometabolic multimorbidity: pooled analysis of individual-level data for 120 813 adults from 16 cohort studies from the USA and Europe. Lancet Public Health. 2017;2: e277–e285. doi:10.1016/S2468-2667(17)30074-9

24.	Tian X, Zuo Y, Chen S, Meng X, Chen P, Wang Y, et al. Distinct triglyceride-glucose trajectories are associated with different risks of incident cardiovascular disease in normal-weight adults. Am Heart J. 2022;248: 63–71. doi:10.1016/j.ahj.2022.02.014

25.	Dang K, Wang X, Hu J, Zhang Y, Cheng L, Qi X, et al. The association between triglyceride-glucose index and its combination with obesity indicators and cardiovascular disease: NHANES 2003–2018. Cardiovasc Diabetol. 2024;23: 8. doi:10.1186/s12933-023-02115-9

26.	Liu Q, Cui H, Ma Y, Han X, Cao Z, Wu Y. Triglyceride-glucose index associated with the risk of cardiovascular disease: the Kailuan study. Endocrine. 2022;75: 392–399. doi:10.1007/s12020-021-02862-3

27.	Feng X, Zhu J, Hua Z, Yao S, Tong H. Comparison of obesity indicators for predicting cardiovascular risk factors and multimorbidity among the Chinese population based on ROC analysis. Sci Rep. 2024;14: 20942. doi:10.1038/s41598-024-71914-1

28.	Klöting N, Fasshauer M, Dietrich A, Kovacs P, Schön MR, Kern M, et al. Insulin-sensitive obesity. Am J Physiol Endocrinol Metab. 2010;299: E506-515. doi:10.1152/ajpendo.00586.2009

29.	Bergeron R, Previs SF, Cline GW, Perret P, Russell RR, Young LH, et al. Effect of 5-aminoimidazole-4-carboxamide-1-beta-D-ribofuranoside infusion on in vivo glucose and lipid metabolism in lean and obese Zucker rats. Diabetes. 2001;50: 1076–1082. doi:10.2337/diabetes.50.5.1076

30.	Xiao D, Sun H, Chen L, Li X, Huo H, Zhou G, et al. Assessment of six surrogate insulin resistance indexes for predicting cardiometabolic multimorbidity incidence in Chinese middle-aged and older populations: Insights from the China Health and Retirement Longitudinal Study. Diabetes/Metabolism Research and Reviews. 2024;40: e3764. doi:10.1002/dmrr.3764
